# Chicken gga-miR-19a Targets ZMYND11 and Plays an Important Role in Host Defense against *Mycoplasma gallisepticu*m (HS Strain) Infection

**DOI:** 10.3389/fcimb.2016.00102

**Published:** 2016-09-14

**Authors:** Qingchang Hu, Yabo Zhao, Zaiwei Wang, Yue Hou, Dingren Bi, Jianjun Sun, Xiuli Peng

**Affiliations:** ^1^Key Laboratory of Agricultural Animal Genetics, Breeding and Reproduction, Ministry of Education, Huazhong Agricultural UniversityWuhan, China; ^2^China National Key Laboratory of Agricultural Microbiology, Huazhong Agricultural UniversityWuhan, China; ^3^Department of Biological Sciences and Border Biomedical Research Center, University of Texas at El PasoEl Paso, TX, USA

**Keywords:** *Mycoplasma gallisepticum* (HS strain), chicken, gga-miR-19a, cell cycle, ZMYND11, NF-κB signaling pathway

## Abstract

*Mycoplasma gallisepticum* (MG), one of the most pathogenic *Mycoplasmas*, can cause chronic respiratory disease (CRD) in chickens. It has been suggested that micro-ribonucleic acids (miRNAs) are involved in microbial pathogenesis. However, little is known about the roles of miRNAs in MG infection. Previously, we found by deep sequencing that gga-miR-19a was significantly up-regulated in the lungs of MG-infected chicken embryos. In this work, we confirmed that gga-miR-19a was up-regulated in both MG-infected chicken embryonic lungs and MG-infected DF-1 (chicken embryo fibroblast) cells. At 72 h post-transfection, we found that the over-expression of gga-miR-19a significantly enhanced the proliferation of MG-infected DF-1 cells by promoting the transition from the G1 phase to the S and G2 phases, while a gga-miR-19a inhibitor repressed the proliferation of MG-infected DF-1 cells by arresting the cell cycle in the G1 phase. Moreover, we found that gga-miR-19a regulated the expression of the host zinc-finger protein, MYND-type containing 11 (*ZMYND11*), through binding to its 3′ untranslated region (3′-UTR). DAVID analysis revealed that *ZMYND11* could negatively regulate the NF-kappaB (NF-κB) signaling pathway in chickens (*Gallus gallus*). Upon MG infection, gga-miR-19a, *NF-*κ*B, MyD88*, and *TNF-*α were all up-regulated, whereas *ZMYND11* was down-regulated. The over-expression of gga-miR-19a in the DF-1 cells did not affect the above gene expression patterns, and gga-miR-19a inhibitor repressed the expression of *NF-*κ*B, MyD88*, and *TNF-*α, but enhanced the expression of *ZMYND11*. In conclusion, gga-miR-19a might suppress the expression of *ZMYND11* in MG-infected chicken embryonic lungs and DF-1 cells, activate the NF-κB signaling pathway, and promote pro-inflammatory cytokines expression, the cell cycle progression and cell proliferation to defend against MG infection.

## Introduction

*Mycoplasma*, an important prokaryote, can infect humans, wildlife, and a wide range of economically important livestock species (Gambarini et al., 2009; Osman et al., 2009; Nicholas and Ayling, 2016). One of the most important *Mycoplasma* species, *Mycoplasma gallisepticum* (MG) is the causative pathogen of avian chronic respiratory disease (CRD) (Ley, 2003), which is invasive and causes severe inflammation in the tracheas and lungs of chickens and turkeys around the world (Davidson et al., 1982; Yoder, 1991; Stipkovits et al., 2012). MG has been shown to invade, survive and multiply inside chicken fibroblasts and HeLa cells (Winner et al., 2000). After infection, MG is very difficult to be eliminated from chicken farms. Although, vaccination and antibiotics can be used to control the infection, it is impossible to completely clear MG from infected chickens. As a consequence, mycoplasmosis is causing huge economic losses to the poultry industry worldwide (Pennycott et al., 2005).

Micro-ribonucleic acids (miRNAs), at 22–25 nucleotides long, are small non-coding single-strand RNAs that negatively regulate gene expression by interfering with post-transcriptional protein translation (Zamore and Haley, 2005; Valencia-Sanchez et al., 2006; O'Reilly, 2016). It has been suggested that up to 30% of human genes are regulated by miRNAs (Di Leva et al., 2006). So far, over 24, 000 (in total) and 859 (in chickens) miRNAs have been identified (http://www.mirbase.org/), but only a few have been functionally studied.

miRNAs play important roles in regulatory pathways, including various physiological and pathological processes (Lim et al., 2003; Ambros, 2004). Emerging data are showing that miRNAs contribute to the development and control of inflammatory responses in both immune and non-immune cells (Bazzoni et al., 2009; Perry et al., 2009).

Current studies suggest that miRNAs are involved in various diseases of poultry, such as avian influenza (Wang et al., 2009), avian leucosis (Wang Q. et al., 2013; Li H. et al., 2014), ovarian carcinoma (Lee et al., 2012), infection bursal disease (Wang Y. S. et al., 2013), and Marek's disease (Yao et al., 2008; Lian et al., 2012; Stik et al., 2013; Li X. et al., 2014). Our previous study has shown that gga-miR-101-3p plays a crucial role in MG infection by regulating EZH2 expression (Chen et al., 2015). The functional study of miRNAs could help to reveal molecular pathways involved in microbial pathogenesis and provide a theoretical basis for miRNA-mediated gene therapy. However, no expression profiles of miRNAs have been reported in MG-infected chickens.

As a major transcriptional factor, nuclear factor-kappaB (NF-κB) regulates genes involved in innate and adaptive immunity, cell proliferation, differentiation and inflammation, especially the rapid response to pathogen infection and pro-inflammatory stimuli (Lindsay, 2008; Yoshida et al., 2013). Activation of the NF-κB signaling cascade results in the expression of pro-inflammatory cytokines and chemokines (Hayden and Ghosh, 2008). miRNAs are also involved in modulating the NF-κB signaling pathway (Lecellier et al., 2005; Ma et al., 2011a; Wendlandt et al., 2012).

miR-19a is up-regulated in a variety of human cancers, including lung cancer (Navarro et al., 2009), colon cancer (Zhang J. et al., 2012), cervical carcinoma (Xu et al., 2012), breast cancer (Zhang et al., 2011), gliomas (Jia et al., 2013), gastric cancer (Wu et al., 2014), and bladder cancer (Feng et al., 2014). miR-19a might regulate the NF-κB signaling pathway in inflammation (Gantier et al., 2012; Ye et al., 2012). Our preliminary deep sequencing data revealed that gga-miR-19a is up-regulated in MG-infected embryonic lungs (unpublished lab data), which suggests that gga-miR-19a might play a crucial role in the response to MG infection. In this study, we further demonstrate that gga-miR-19a is significantly up-regulated in MG-infected chicken embryonic lungs and DF-1 cells. Furthermore, we identified ZMYND11 as a gga-miR-19a target and followed up with a detailed miRNA regulation of ZMYND11 expression, the cell cycle, cell proliferation and the NF-κB signaling pathway in the context of MG infection.

## Materials and methods

### Ethics statement

Our experimental protocols for chicken-embryo treatment were approved by the Institutional Animal Care and Use Committee of Huazhong Agricultural University. The procedures were carried out in accordance with the approved guidelines.

### gga-miR-19a target analysis

Potential gga-miR-19a targets were predicted by TargetScan (http://www.targetscan.org/) and miRDB (http://www.mirdb.org/miRDB/). The duplex and minimum free energy (mFE) between gga-miR-19a and 3′-UTR of its potential targets were estimated by RNA hybrid (http://bibiserv.techfak.uni-bielefeld.de/rnahybrid/). The conservation of the target gene was analyzed by TargetScan (http://www.targetscan.org/). The functions of the target genes of gga-miR-19a in chickens were analyzed using DAVID Bioinformatics Resources 6.7 (http://david.abcc.ncifcrf.gov/).

### Design of DNA primers and synthesis of RNA oligonucleotides

The sequences of all the primers used in this study are listed in Table 1. The sequences of RNA oligonucleotides are shown in Table 2. A gga-miR-19a mimics (denoted as miR-19a) and an inhibitor (denoted as miR-19a-Inh) were designed and synthesized by GenePharma (Shanghai, China). A random miRNA mimics that had not been found to suppress any chicken target genes (denoted as miR-19a-NC), and a random miRNA inhibitor that had not been found to promote any chicken target genes (denoted as miR-19a-Inh-NC) were also designed and synthesized to serve as the negative controls.

**Table 1:** **Sequences of DNA primers**.

**Name**	**Primer sequence (5′-3′)**	**Accession no**.
**PRIMERS FOR 3′-UTR CLONING**		
ZMYND11 3′-UTR-F	TTCCTCGAGTAGAACATCAC	XM-004939164
ZMYND11 3′-UTR-R	AATGCGGCCGCCATCAAGGTAGTATGCTTCGTTT	XM-004939164
**PRIMERS FOR RT-qPCR**		
GAPDH-F	GAGGGTAGTGAAGGCTGCTG	NM-204305
GAPDH-R	CACAACACGGTTGCTGTATC	NM-204305
RT- gga-miR-19a-3p	CTCAACTGGTGTCGTGGAGTCGGCAATTCAGTTGAGTCAGTTTT	MIMAT-0001112
gga-miR-19a-3p-F	CTGGTAGGTGTGCAAATCCATG	MIMAT-0001112
gga-miR-19a-3p-R	GGTGTCGTGGAGTCGGCAAT	MIMAT-0001112
gga-5s-rRNA-F	CCATACCACCCTGGAAACGC	
gga-5s-rRNA-R	TACTAACCGAGCCCGACCCT	
ZMYND11-F	ACCAGCGATTCCTTCGTGAG	XM-004939164
ZMYND11-R	TGGGCTGAGGCATTGTGGGA	XM-004939164
MyD88-F	TCAGTTTGTCCAGGAGATG	NM-001030962
MyD88-R	GGTGTAATGAACCGCAAGATA	NM-001030962
NF-κB-F	GCCAGGTTGCCATCGTGT	NM-205129
NF-κB-R	CGTGCGTTTGCGCTTCTC	NM-205129
TNF-α-F	GGACAGCCTATGCCAACAAG	XM-015294124
TNF-α-R	ACACGACAGCCAAGTCAACG	XM-015294124

**Table 2:** **Sequences of RNA oligonucleotides**.

**Name**	**Sequences (5′-3′)**
gga-miR-19a mimics	UGUGCAAAUCUAUGCAAAACUGA AGUUUUGCAUAGAUUUGCACAUU
gga-miR-19a NC sense	UUCUCCGAACGUGUCACGUTT
gga-miR-19a NC antisense	ACGUGACACGUUCGGAGAATT
gga-miR-19a inhibitor	UCAGUUUUGCAUAGAUUUGCACA
gga-miR-19a inhibitor NC	CAGUACUUUUGUGUAGUACAA

### *Mycoplasma* strains and cell culture

MG-HS is a virulent strain isolated from a chicken farm in Hubei province, China (Bi and Ji, 1988; Bi and Xu, 1997). The strain was deposited and donated by the State Key Laboratory of Agricultural Microbiology, College of Veterinary Medicine, Huazhong Agricultural University (Wuhan, Hubei 430070, China). MG-HS culture and concentration determination were carried out as previously described (Bi and Ji, 1988), and its viable number in suspension was detected by a color-changing unit (CCU) assay (Calus et al., 2010).

The immortalized chicken embryonic fibroblast cell line DF-1 was obtained from the American Type Culture Collection (Rockville, MD, USA) and cultured in Dulbecco's modified Eagle's medium (DMEM, Invitrogen, Carlsbad, CA, USA) supplemented with 10% fetal bovine serum (FBS, Invitrogen Gibco Co., Carlsbad, CA, USA), 100 units of penicillin G and 100 μg of streptomycin (Beyotime Institute of Biotechnology, Haimen, China) per milliliter, at 39°C in a humidified 5% CO_2_ incubator.

### gga-miR-19 target dual-luciferase reporter assay

To construct the dual luciferase reporter plasmid, the 3′-UTR region of ZMYND11 covering the predicted gga-miR-19a binding site was amplified by RT-PCR using the cDNA extracted from the chicken embryo lung tissues as the template. The amplified fragment was sub-cloned into the *Xho* I/*Not* I sites of the psi-CHECK™-2 vector (Promega, Madison, WI, USA). See Table 1 for primer sequences. All PCR products were confirmed by sequencing.

DF-1 cells were plated in 24-well plates at 2 × 10^5^ cells per well for the luciferase assay. Next, 200 ng of the luciferase reporter plasmid and 10 pmol of miR-19a, miR-19a-NC, miR-19a-Inh or miR-19a-Inh-NC were also transfected into DF-1 cells using Lipofectamine 2000 (Invitrogen, Carlsbad, CA, USA). The cells were collected at 48 h post-transfection, and the dual-luciferase activity was measured using a Lumat LB 9507 Ultra Sensitive Tube Luminometer (Titertek Berthold, Nanjing, People's Republic of China) according to the manufacturer's protocol (Promega, USA). The firefly luciferase activity of each sample was normalized to the Renilla luciferase activity. Three independent repeats were performed for all above transfection experiments.

### Infection experiments

DF-1 cells were detached from cell culture vials by trypsin treatment, evenly plated in six-well plates and then incubated in the medium without antibiotics. The MG-infection experiments were divided into an experimental group and a control group, and each infection experiment was repeated three independent times with three replicates of each sample. When the cells in the experimental group reached 80–90% confluence, they were infected with 100 μl of MG at the mid-exponential phase (1 × 10^10^ CCU/ml). The cells in both groups were collected in Trizol (Invitrogen, Carlsbad, CA, USA) for further use at 24 h post-infection. Infected-chicken embryonic lung tissues and the controls were deposited in Key Laboratory of Agricultural Animal Genetics, Breeding and Reproduction, Ministry of Education, Huazhong Agricultural University (Wuhan, Hubei 430070, China).

### Quantitative PCR

Total RNA was extracted from the cultured cells or the frozen chicken embryonic lung tissues with TRIzol Reagent (Invitrogen, Carlsbad, CA, USA). The purification of RNA was performed using RNeasy mini columns according to the manufacturer's protocol (Qiagen; Valencia, CA). Using the Prime Script™ RT reagent kit with gDNA eraser (TaKaRa, Tokyo, Japan), RT-qPCR was performed using 1 μg of total RNA from each sample with TransStart Top Green qPCR SuperMix (TRANSGEN, Beijing, China) on the CFX96 or CFX384 TouchTM (Bio-Rad, Hercules, CA, USA). The Ct (2^−ΔΔCt^) method was used to calculate relative expression of gga-miR-19, *ZMYND11, NF-*κ*B, MyD88*, and *TNF-*α (Livak and Schmittgen, 2001). The data were analyzed using 7500 software v.2.0.1 (Applied Biosystems, Foster City, CA, USA), with the automatic Ct used to determine the baseline and threshold for Ct determination. 5S-RNA was used as an internal control for miR-19a, and glyceraldehyde-3-phosphate dehydrogenase (GAPDH) was used as an internal control for *ZMYND11, NF-*κ*B, TNF-*α and *MyD88*. The primers are listed in Table 1. The experiment was performed three independent times with three replicates of each sample, for a total of nine samples.

DF-1 cells were seeded in six-well plates at 6 × 10^3^ cell/well in 100 μl of DMEM containing 10% (v/v) FBS and incubated overnight at 39°C with 5% CO_2_. Before transfection, the DF-1 cells were washed twice using phosphate-buffered saline (PBS), and Opti-MEMI ReduMced Serum was added. Firstly, 200 μl of Opti-MEMI ReduMced Serum and 6 μl of Lipofectamin 2000 (Invitrogen Life Technologies, USA) were added to centrifuge tube A. Next, 7.5 pmol of miR-19a, miR-19a-NC, miR-19a-Inh or miR-19a-Inh-NC, and 200 μl of Opti-MEMI ReduMced Serum were added to tube B and reacted for 5 min at room temperature. Next, tubes A and B were mixed for 20 min at room temperature. The mixture of tubes A and B was added into six-well plates and continuously incubated for 4 h with DMEM containing 10% (v/v) FBS, 100 IU/ml penicillin G and 100 μg/ml streptomycin to replace the previous medium. In addition, a mock transfection was used as a blank control (denoted as blank). The cells were collected at 48 h post-transfection. Total RNA was extracted and purified, and RT-PCR was performed in a TransStart Top Green qPCR SuperMix (TRANSGEN, China) on CFX96 or CFX384 TouchTM (Bio-Rad, USA). The transfections were performed three independent times. The relative mRNA levels of *NF-*κ*B, MyD88* and *TNF-*α were calculated using the 2^−ΔΔCt^ method (Livak and Schmittgen, 2001).

### Western blot analysis

DF-1 cells were seeded in 24-well plates and transfected with the indicated RNA oligonucleotides. Total proteins were isolated from the DF-1 cells 48 h post-transfection using RIPA-buffer (Beyotime, Beijing, China) with 100 mM phenylmethanesulfonyl fluoride (PMSF). Protein concentrations were determined with the bicinchoninic acid (BCA) protein assay reagent kit (Beyotime, Beijing, China). Then, 10 μg of the total protein was separated using 12% sodium dodecylsulfate-polyacrylamide gel electrophoresis (SDS-PAGE) and transferred to polyvinylidene fluoride (PVDF) membranes (Beyotime, Beijing, China) by electrophoresis for 2 h at 80 mA. Membranes were blocked with 5% (w/v) fat-free milk at room temperature for 1 h. The membrane was incubated overnight with primary antibodies at 4°C, including goat polyclonal anti-ZMYND11 (Santa Cruz, Biotechnology, Inc. Santa Cruz, CA, USA) and rabbit anti-β-actin, which served as a protein loading control. Then, the membrane was washed and incubated with rabbit anti-goat secondary antibody for 1 h. After three washes with TBST, antigen-antibody complexes on the membranes were detected using an enhanced chemiluminescence (ECL) detection system (Bio-Rad, Hercules, CA, USA). All assays were performed in triplicate.

### Cell proliferation and cell cycle assays

A total of 6 × 10^3^ DF-1 cells were plated in triplicate in six-well plates in 100 μl of DMEM medium containing 10% (v/v) FBS and incubated overnight at 39°C in a humidified 5% CO_2_ incubator. DF-1 cells were then transfected with miR-19a, miR-19a-NC, miR-19a-Inh or miR-19a-Inh-NC, as described above. At 4 h post-transfection, the cells then were infected with 7 μl of MG-HS strain at 10^10^ CCU/ml. At 24 h, 48 h, 72 h post-transfection, 10 μl of the CCK-8 solution was added to each well of the plate, which was then incubated at 39°C for 4 h. The infected-MG cells (denoted as miR-free MG+) and the uninfected-MG cells (blank MG-) were used as controls. The blank MG-cells were cultured in a sterile incubator to avoid MG contamination. Cell proliferation was determined using the Cell Counting Kit-8 according to the manufacturer's protocol (CCK-8, DOJINDO, Shanghai, China). The optical density at 450 nm of each well plate was measured using a microplate reader (Bio-Rad, Hercules, CA, USA).

The cell cycle assay was performed in 24-well plates. The DF-1 cells were transfected with gga-miR-19a, miR-19a-NC, miR-19a-Inh or miR-19a-Inh-NC. At 4 h post-transfection, the cells were infected with 7 μl of MG-HS strain at 10^10^ CCU/ml. At 48 h post-transfection, the cells were harvested, washed with ice-cold PBS, and fixed in ice-cold 70% ethanol in PBS for 12 h. Similarly, miR-free-MG+ and blank MG− were used as controls. The cell cycle was analyzed with a flow cytometer, using the cell cycle detection kit (KeyGEN, Nanjing, China). The percentages of the cells in the G1, S and G2 phases were calculated. Three replicate wells were included in each experimental group, and all experiments were repeated independently in triplicate three separate times.

### Statistical analysis

All assays were performed three times in triplicate, and all nine values were applied to statistical analysis. The data are presented as the mean ± SD and were analyzed by Student's *t*-test. A *p* < 0.05 was considered statistically significant (^*^P < 0.05, ^**^P < 0.01).

## Results

### Prediction of the target gene of gga-miR-19a

Using miRNA deep sequencing, we previously found that gga-miR-19a was up-regulated in infected chicken embryonic lung tissues (unpublished lab data).

**Figure 1:**
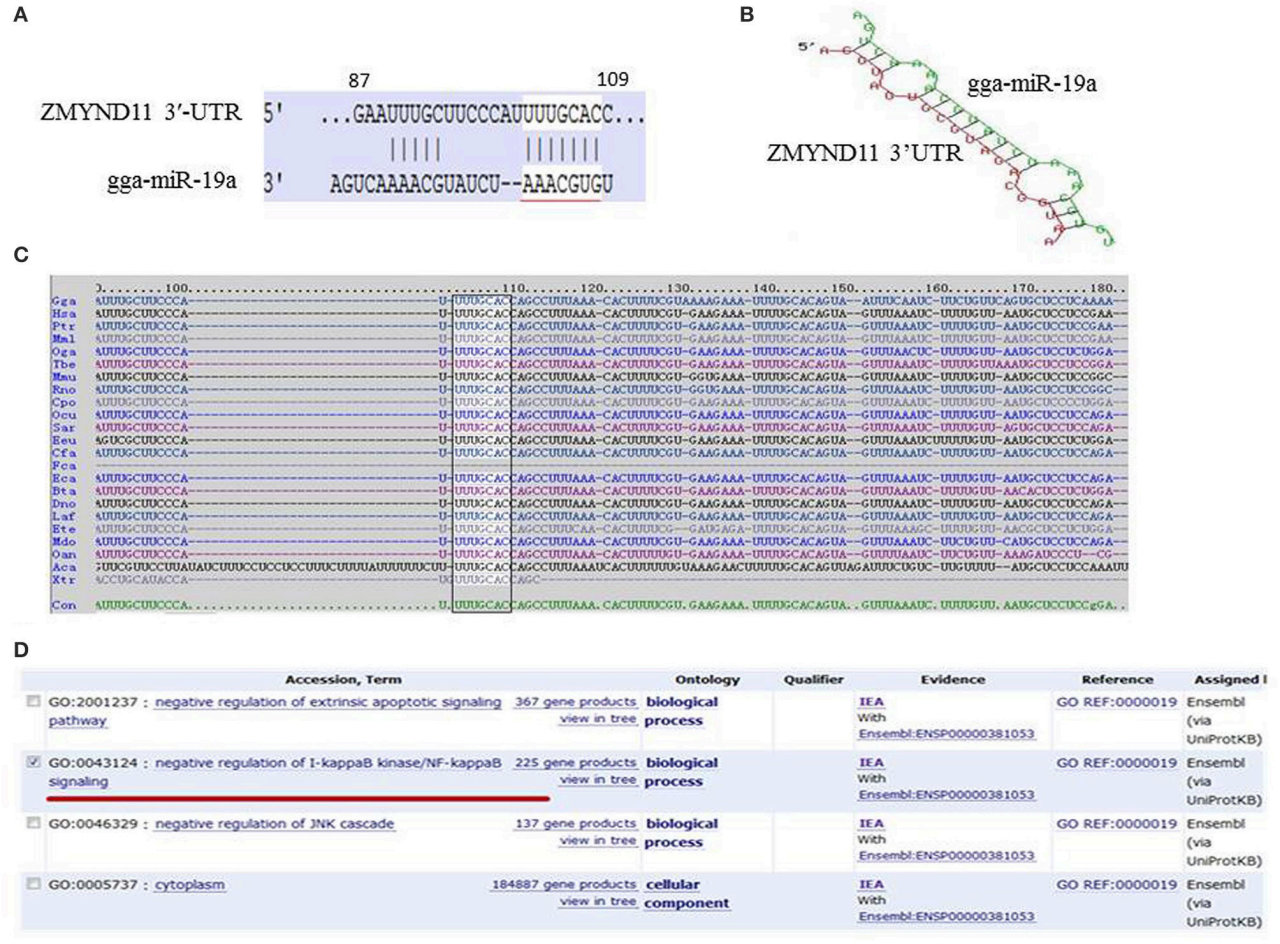
**Bioinformatics analysis of the target genes of gga-miR-19a. (A)** Sequence schematic diagram of gga-miR-19a and the target site in the 3′-UTR of *ZMYND11*. The seed sequence of gga-miR-19a is underlined, and the matching or complementary nucleotides between gga-miR-19a and *ZMYND11* 3′-UTR are indicated; **(B)** The predicted secondary structure of the RNA duplex of gga-miR-19a and the *ZMYND11* 3′-UTR target site (Red: Target sequence; Green: gga-miR19a); **(C)** Sequence alignment of *ZMYND11* 3′-UTR from different species. The conserved target sequences are highlighted; **(D)** Predicted function of the target gene of gga-miR-19a.

To identify the targets of gga-miR-19a, we carried out a bioinformatics analysis using targetScan (http://www.targetscan.org/) and miRDB (http://www.mirdb.org/miRDB/). *ZMYND11* was listed as a potential gga-miR-19a target gene based on its high score (score 98), and the highly conserved target site on the 3′-UTR of *ZMYND11* in a wide range of species, including human, mouse, dog, monkey, platypus, etc. (Figure 1C). The predicted target site is a 87–109 base sequence, and the potential binding site is a 102–108 base sequence (Figure 1A). The minimum free energy (mFE) between *ZMYND11* 3′-UTR and gga-miR-19a was calculated in RNAhybrid software (http://bibiserv.techfak.uni-bielefeld.de/rnahybrid/). The mFE was about −20.5 kCal/mol, which indicates high stability (Figure 1B). The functions of ZMYND11 in chickens were investigated using DAVID Bioinformatics Resources 6.7 (http://david.abcc.ncifcrf.gov/) (Ikeda et al., 2010), which suggests negative regulation of the NF-κB signaling pathway (Figure 1D).

### *ZMYND11* is the direct target of gga-miR-19a

**Figure 2:**
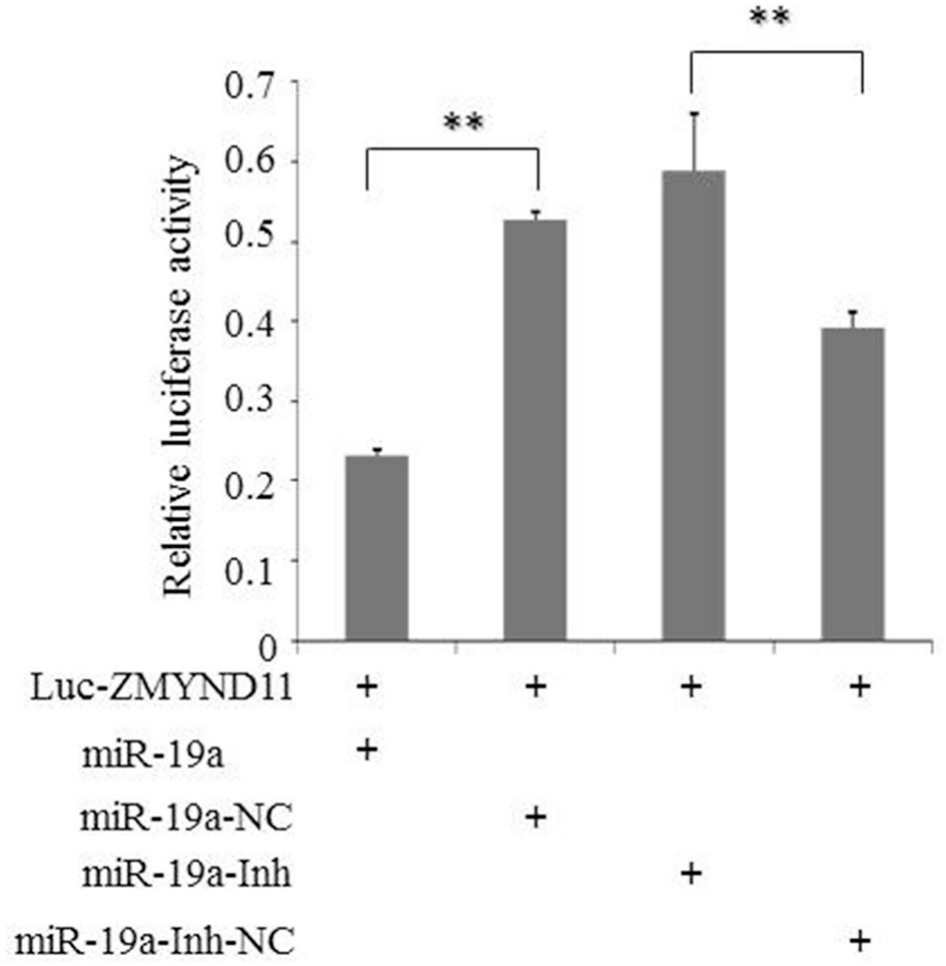
**Gga-miR-19a directly targets to *ZMYND11***. DF-1 cells were co-transfected with Luc- *ZMYND11* (3′-UTR) and the indicated RNA oligonucleotides. Luciferase activity was assayed at 24 h post-transfection. The firefly luciferase activity of each sample was normalized to the Renilla luciferase activity. All values are represented as the mean ± SD of three independent experiments in triplicate and were analyzed by Student's *t*-test. Significant differences are denoted as ^**^P < 0.01.

To further clarify whether gga-miR-19a directly targets to the 3′-UTR of *ZMYND11*, we used a luciferase reporter gene assay (psi-CHECK™-2), where the firefly luciferase gene was fused to the entire 3′-UTR of *ZMYND11* (Luc-*ZMYND11*), and the Renilla luciferase was used for normalization. A luciferase activity assay was performed 48 h after co-transfection of DF-1 cells with gga-miR-19a mimics (miR-19a) and the Luc-*ZMYND11* (3′-UTR) vector. *ZMYND*11 3′-UTR luciferase activity was significantly inhibited by gga-miR-19a, whereas no effect on luciferase activity was observed in the miR-19a-NC-transfected cells (Figure 2). However, when miR-19a-Inh was transfected into the DF-1 cells, the luciferase activity was significantly increased, even in the presence of gga-miR-19a. As expected, miR-19a-Inh-NC did not have effects on *ZMYND11* 3′-UTR luciferase activity (Figure 2). Altogether, these results indicate that gga-miR-19a inhibited *ZMYND11* gene expression by directly binding to its complementary sequence in the 3′-UTR of *ZMYND11* in a sequence-specific manner.

### gga-miR-19a negatively regulates ZMYND11 expression

**Figure 3:**
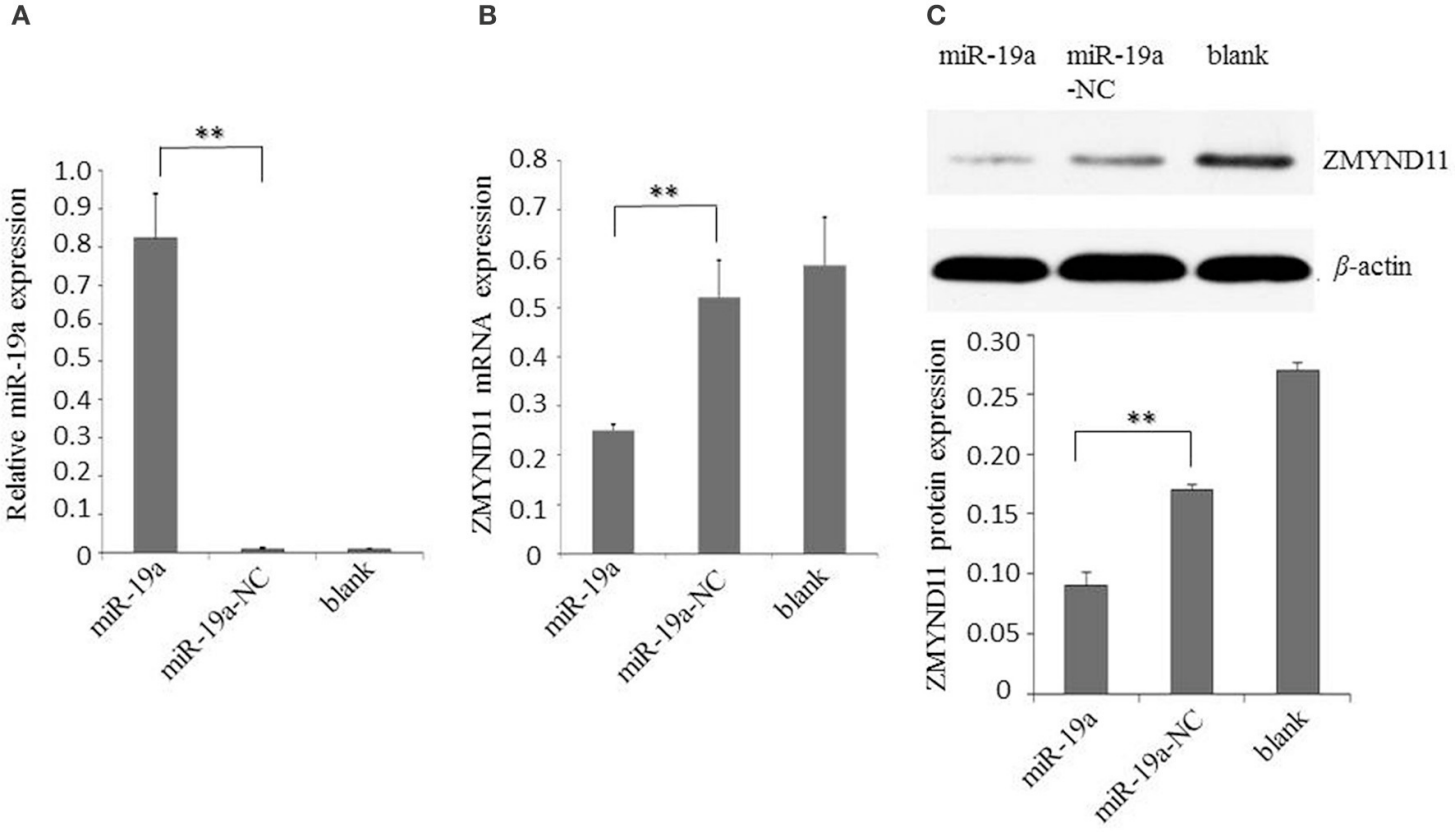
**Gga-miR-19a inhibits ZMYND11 expression. (A)** Over-expression of gga-miR-19a in DF-1 cells; **(B)** The level of ZMYND11 mRNA in DF-1 cells over-expressing gga-miR-19a was determined by RT-qPCR; **(C)** Western blot was performed to analyze ZMYND11 protein expression in DF-1 cells at 48 h post- transfection with gga-miR-19a. A mock transfection was used as the blank. GAPDH was used as an internal quantitative control. All values are represented as the mean ± SD of three independent experiments in triplicate. Significant differences are denoted as ^**^P < 0.01.

**Figure 4:**
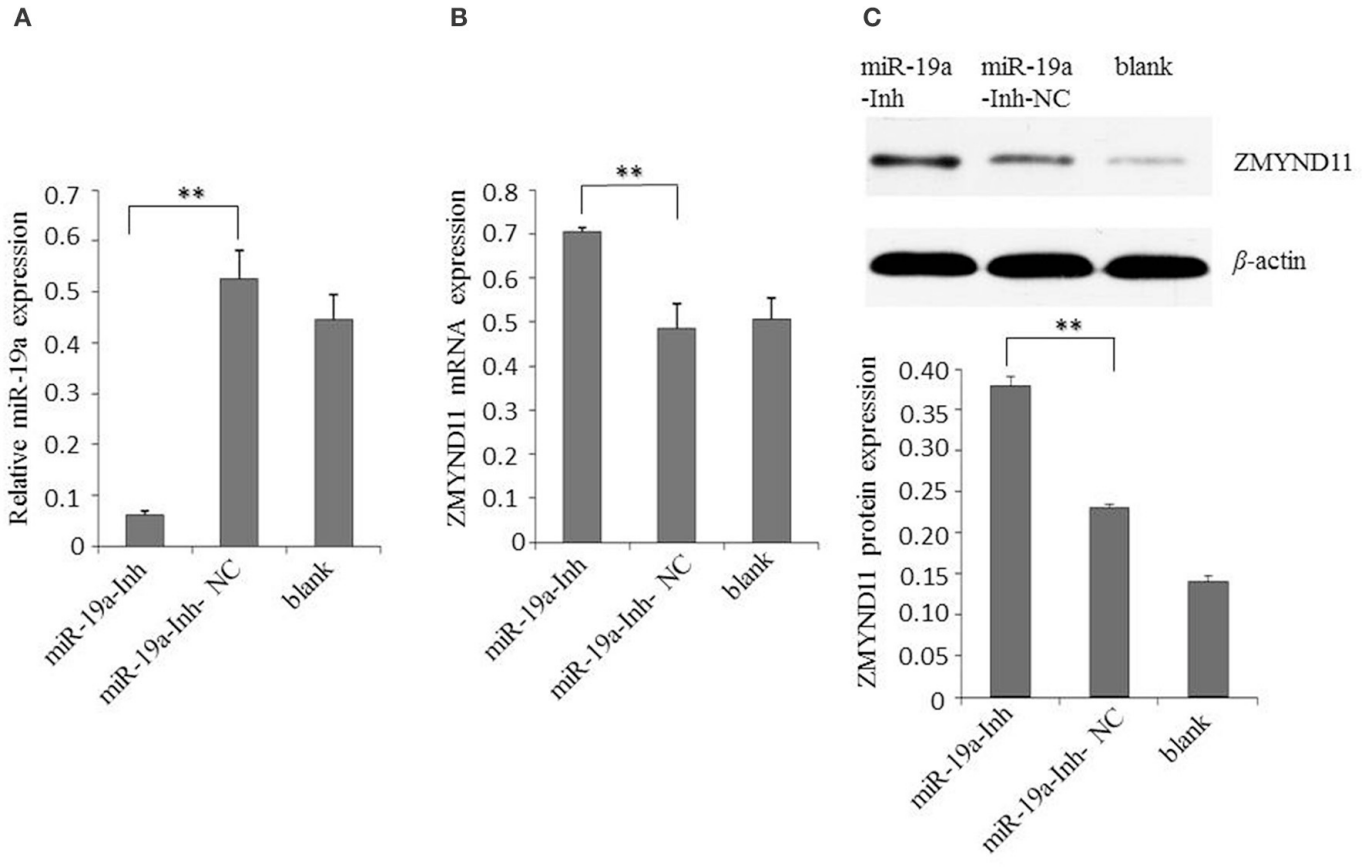
**Inhibition of gga-miR-19a increases ZMYND11 expression. (A)** Transfection of miR-19a-Inh reduced the expression of gga-miR-19a in DF-1 cells; **(B)** Transfection of miR-19a-Inh increased the expression of ZMYND11 in DF-1 cells; **(C)** Western blot was performed to analyze ZMYND11 protein expression in DF-1 cells at 48 h post-transfection with miR-19a-Inh. A mock transfection was used as the blank; the expression of GAPDH was used as a loading control. All values are represented as the mean ± SD of three independent experiments in triplicate. Significant differences are denoted as ^**^P < 0.01.

Next, we examined the effect of gga-miR-19a on endogenous ZMYND11 expression in detail. By using qPCR and a Western blot, we studied the expression of ZMYND11 in DF-1 cells that were transfected with miR-19a, miR-19a-NC, miR-19a-Inh or miR-19a-Inh-NC. By transient transfection, gga-miR-19a was over-expressed in DF-1 cells at 88.9 times the level of endogenous gene expression (miR-19a-NC) (Figure 3A). gga-miR-19a mimics significantly decreased ZMYND11 expression at both the mRNA level and protein levels at 48 h post-transfection (Figures 3B,C). In contrast, the endogenous gga-miR-19a was reduced 8.5-fold in the cells transfected with gga-miR-19a inhibitor (Figure 4A), which led to the increased expression of ZMYND11 at both the mRNA and protein levels at 48 h post-transfection (Figures 4B,C). These results indicate that gga-miR-19a down-regulates ZMYND11 expression through binding to the *ZMYND11* 3′-UTR in DF-1 cells.

### gga-miR-19a promotes the proliferation of MG-infected DF-1 cells by inducing the G1 phase transition into the S and G2 phases

**Figure 5:**
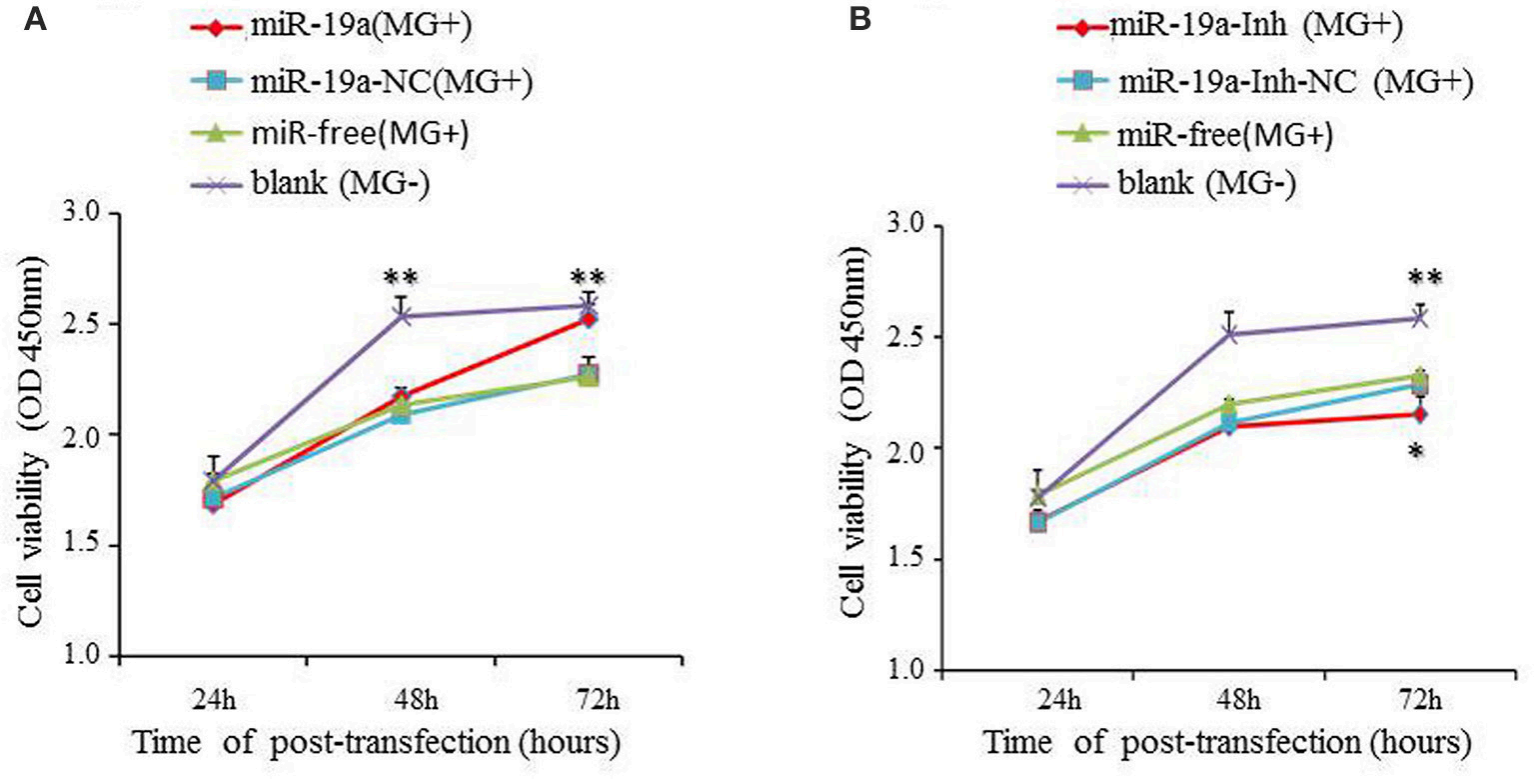
**The effect of gga-miR-19a on DF-1 cell proliferation**. DF-1 cells were transfected with gga-miR-19a, miR-19a-NC, miR-19a-Inh or miR-19a-Inh-NC and were incubated for 4 h. The cells were then infected with the MG-HS strain. Four control groups, including miR-19a-NC (MG+), miR-19a-Inh-NC (MG+), miR-free (MG+) and the blank (MG−), were used. At 24, 48, and 72 h post-transfection, cell proliferation was detected using CCK-Cell Counting Kit-8a. All values are represented as the mean ± SD of three independent experiments in triplicate. The asterisks represented statistically significant differences (^*^P < 0.05, ^**^P < 0.01). **(A)** The over-expression of gga-miR-19a dramatically promotes DF-1 cell proliferation; **(B)** Inhibitor of gga-miR-19a inhibites the proliferation of DF-1 cells.

To elucidate the biological significance of gga-miR-19a in MG-HS pathogenicity, we transfected DF-1 cells with gga-miR-19a mimics and then infected the cells with 7 μl of MG-HS strain at 10^10^ CCU/ml [denoted as miR-19a (MG+)]. In parallel, we prepared three control groups. One was transfected with miR-19a-NC and then infected with 7 μl of MG-HS strain at 10^10^ CCU/ml [denoted as miR-19a-NC (MG+)]; the second was infected with only 7 μl of MG-HS strain at 10^10^ CCU/ml [denoted as miR-free (MG+)]; and the third group consisted of the uninfected DF-1cells [denoted as blank (MG−)]. The proliferation of DF-1 cells was measured at 24, 48, and 72 h post-transfection using the Cell Counting Kit-8. During the first 48 h post-transfection, a decrease in cell viability was observed in all MG-infected groups (including miR-19a-NC, miR-free and over-expressed miR-19a) compared to the blank MG- group. At 72 h post-transfection, although the cell viability of the two control groups, miR-19a-NC (MG+) and miR-free (MG+), remained low, a remarkable increase in DF-1 cell proliferation was observed in the cells over-expressed gga-miR-19a. As a result, the cell viability of the blank MG- group and the test group over-expressing gga-miR-19a (MG+) reached the same level at 72 h post-transfection (Figure 5A). Next, we studied the effects of miR-19a inhibitor on cell proliferation. After miR-19a inhibitor was transfected into DF-1 cells (miR-19a-Inh), the expression of gga-miR-19a was significantly repressed. As expected, this change resulted in a significant reduction in the proliferation of DF-1 cells at 72 h post-transfection, compared to proliferation in the blank MG- group. We also observed a significant reduction in proliferation compared to the miR-19a-Inh-NC (MG+) group or the miR-free group (MG+) (Figure 5B). In summary, our results suggest that gga-miR-19a enhances DF-1 cell proliferation by inhibiting MG propagation.

**Figure 6:**
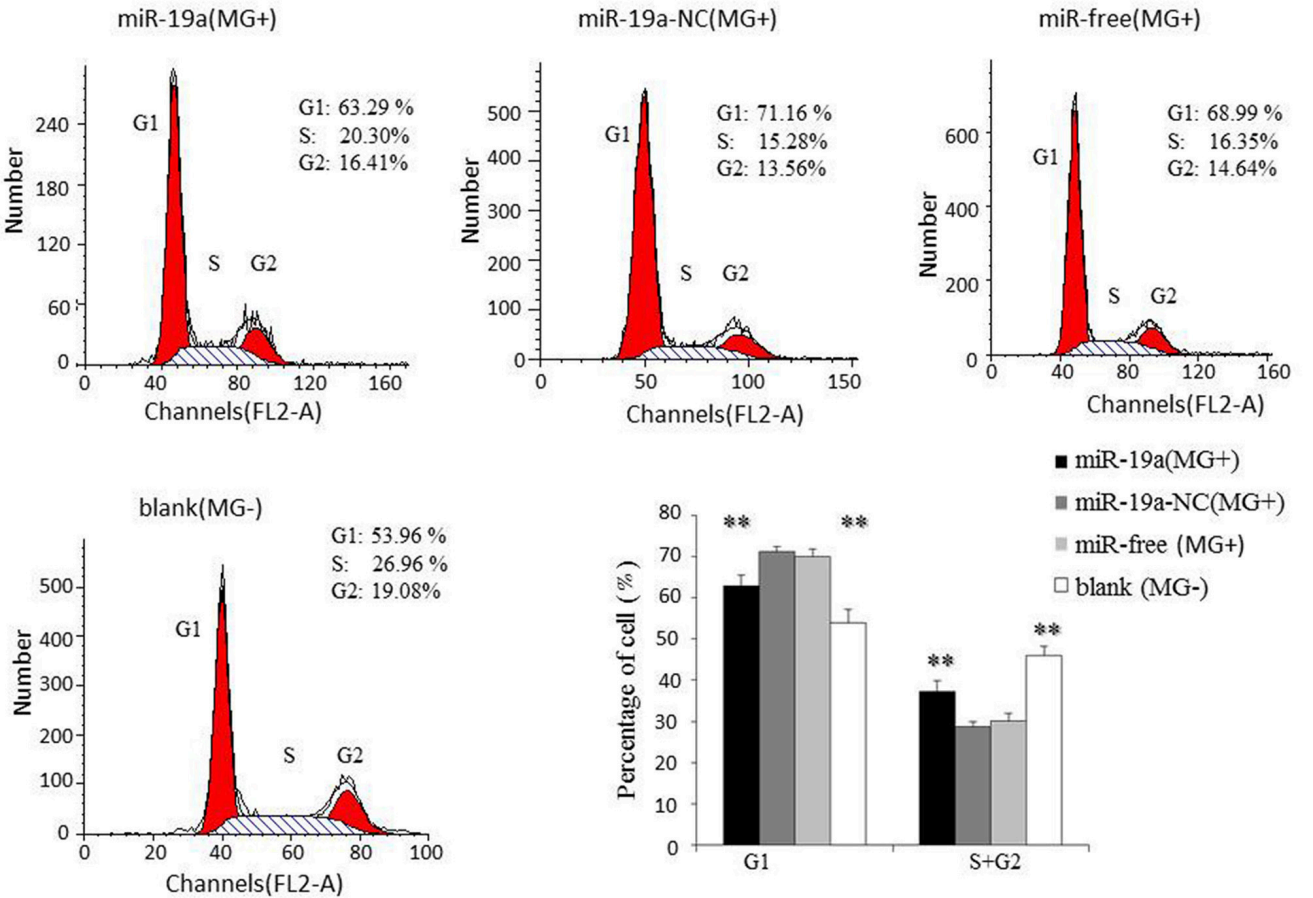
**The effect of the over-expression of gga-miR-19a on the distribution of DF-1 cells in the cell cycle**. DF-1 cells were transfected with gga-miR-19a or miR-19a-NC and were incubated for 4 h. The cells then were infected with MG-HS strain. Three control groups, including miR-19a-NC (MG+), miR-free (MG+) and blank (MG−), were used. At 48 h post-transfection, the cell phase distribution was analyzed using a flow cytometer. Three independent experiments were performed in triplicate. All values are represented as the mean ± SD. Significant differences are denoted as ^**^P < 0.01.

**Figure 7:**
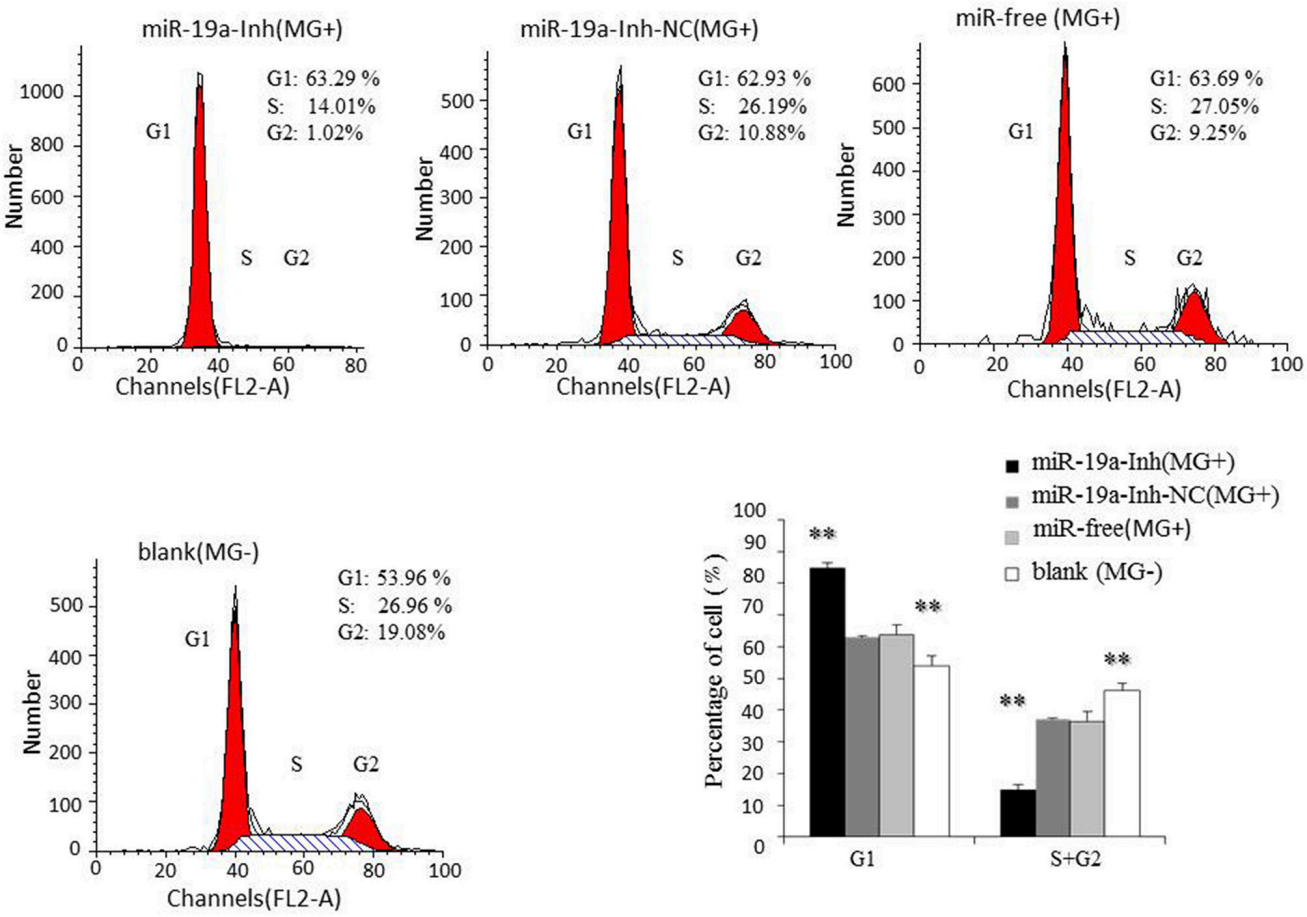
**The effect of gga-miR-19a inhibitor on the distribution of DF-1 cells in the cell cycle**. DF-1 cells were transfected with miR-19a-Inh or miR-19a-Inh-NC and were incubated for 4 h. The cells were then infected with MG-HS strain. Three control groups, including miR-19a-Inh-NC (MG+), miR-free (MG+) and blank (MG−), were used. At 48 h post-transfection, the cell cycle was analyzed using a flow cytometer. All values are represented as the mean ± SD of three independent experiments in triplicate. Significant differences are denoted as ^**^P < 0.01.

To further investigate how gga-miR-19a regulates DF-1 cell proliferation, the distribution of cells in different stages of the cell cycle was assayed with a flow cytometer. Similarly, the synthetic RNA oligonucleotides were transfected into DF-1 cells. MG infection inhibited mitosis by inducing the G1 cell cycle arrest in the DF-1 cells. The over-expression of gga-miR-19a significantly increased the percentage of the S and G2 phases cells, whereas the percentage of the G1 phase cells was significantly decreased compared to that in the control groups except for the blank (MG-) (Figure 6). On the contrary, gga-miR-19a-Inh arrested the cell cycle progression at the G1 phase (Figure 7).

Taken together, these results indicate that gga-miR-19a inhibits MG propagation, and promotes the proliferation of DF-1 cells by affecting the cell cycle.

### Expression of gga-miR-19a, *ZMYND11, NF-κB, TNF-α*,and *MyD88*in MG-infected DF-1 cells and chicken embryonic lungs

**Figure 8:**
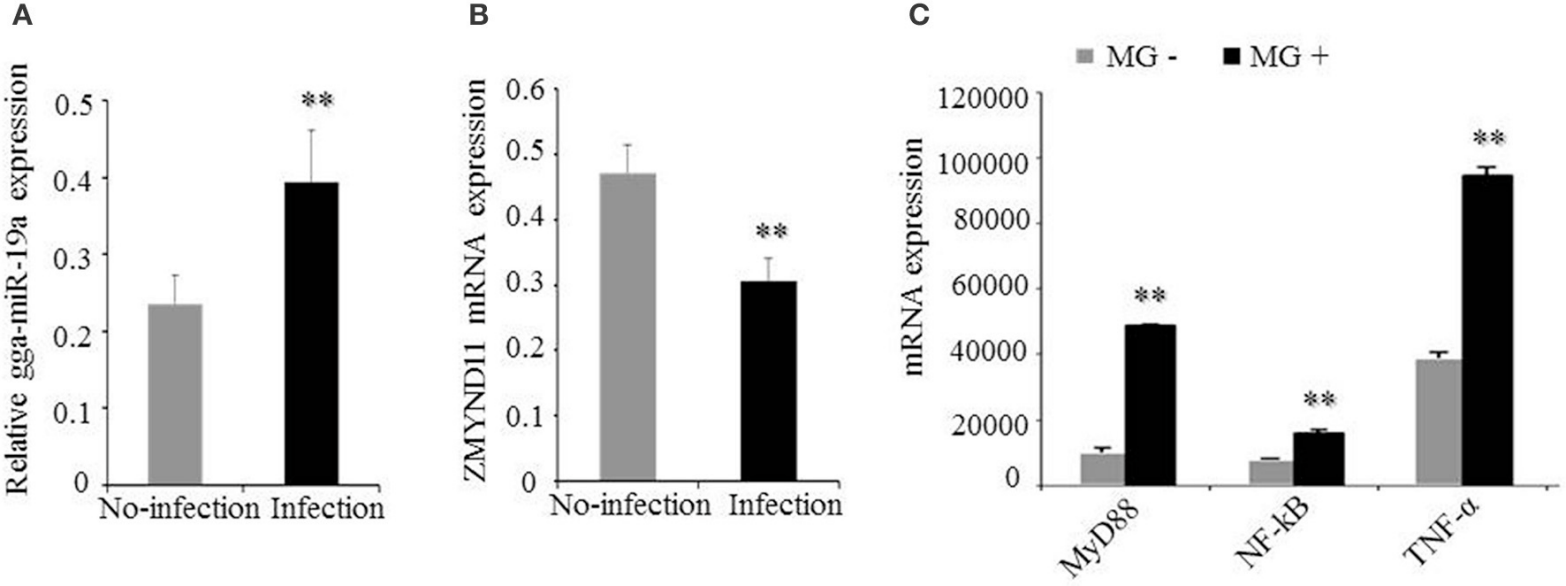
**Effects of MG infection on the expression of gga-miR-19a, *ZMYND11, NF-*κ*B, TNF-*α, and *MyD88* in DF-1 cells**. The DF-1 cells were infected with MG-HS as described in the Material and Methods, and the total RNA was extracted. **(A)** gga-miR-19a expression was assessed by RT-qPCR, using 5S-rRNA as an internal quantitative control; **(B)** *ZMYND11* mRNA expression was assessed by RT-qPCR, using GAPDH as an internal quantitative control; **(C)** *NF-*κ*B, TNF-*α and *MyD88* mRNA expression was assessed by RT-qPCR, using GAPDH as an internal quantitative control. All values are represented as the mean ± SD of three independent experiments in triplicate. Significant differences are denoted as ^**^P < 0.01.

It was shown that *ZMYND11* could negatively regulate the NF-κB signaling pathway in chickens by DAVID analysis (Figure 1D). We demonstrated that gga-miR-19a was significantly up-regulated in MG-infected DF-1 cells relative to its expression in non-infected DF-1 cells (Figure 8A), whereas the expression of *ZMYND11* mRNA was significantly down-regulated (Figure 8B). As expected, the expression of *NF-*κ*B, TNF-*α and *MyD88* were also significantly up-regulated (Figure 8C).

**Figure 9:**
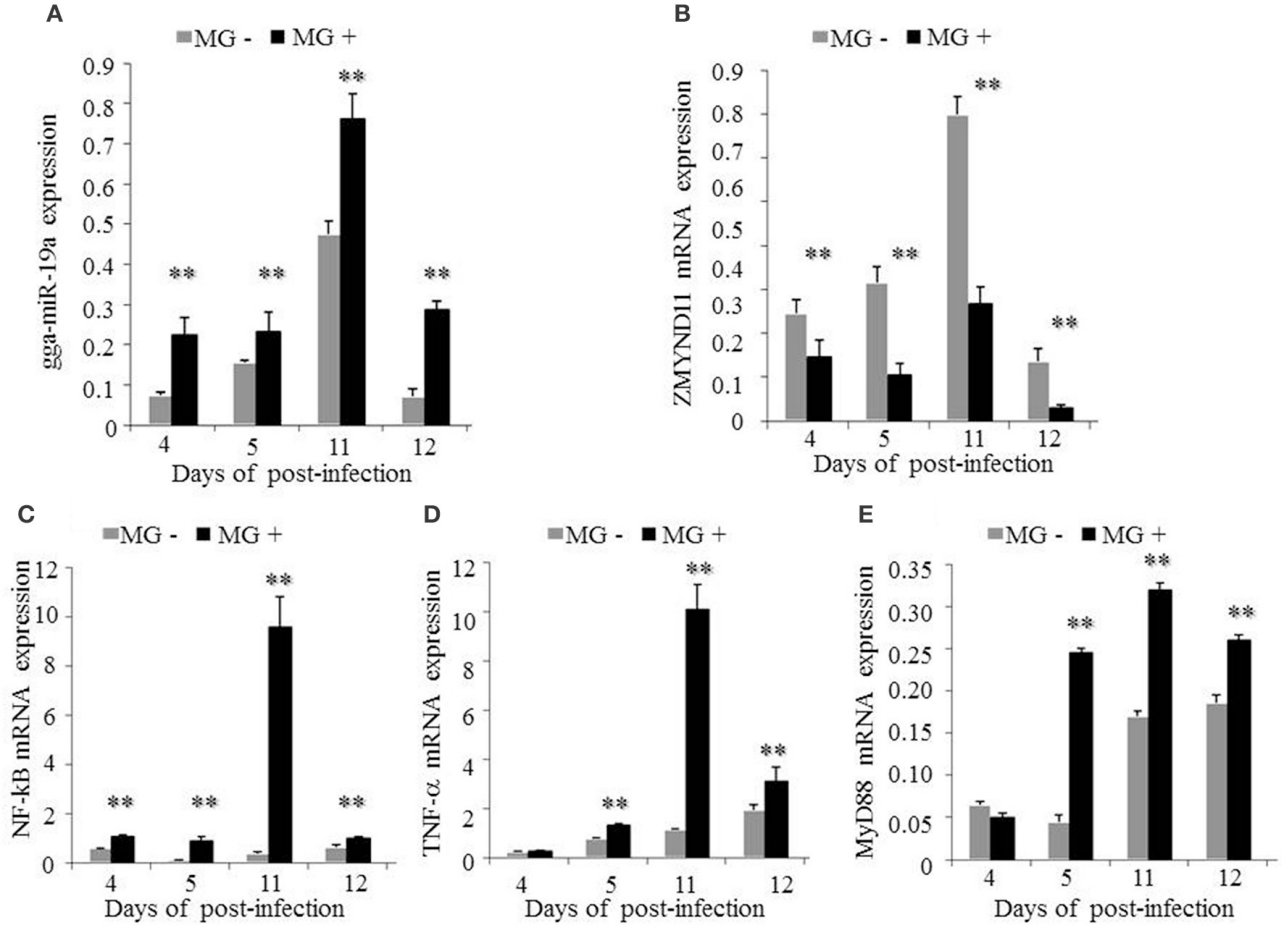
**Effects of MG infection on the expression of gga-miR-19a, *ZMYND11, NF-*κ*B, TNF-*α and *MyD88* in the lungs of chicken embryos**. The chicken embryos were infected by MG-HS as described in the Material and Methods. On the 4, 5, 11, and 12th days post-infection, the lungs of infected chicken embryos were processed, and the expression of gga-miR-19a **(A)**, *ZMYND11* **(B)**, *NF-*κ*B* **(C)**, *TNF-*α **(D)**, and *MyD88* **(E)** were measured by RT-qPCR. All samples were normalized to GAPDH. All values are represented as the mean ± SD of three independent experiments in triplicate. Significant differences are denoted as ^**^P < 0.01.

Subsequently, we examined the effects of MG infection on the expression of the above genes *in vivo* by injecting the chicken embryos with MG-HS on the 9th hatching day. On the 4, 5, 11, and 12th days post-infection (equivalent to the 12, 13, 19, and 20th days of egg hatching), the expression of gga-miR-19a was significantly higher in MG-infected chicken embryonic lungs (Figure 9A). As expected, the expression of *ZMYND11* showed a pattern opposite that of gga-miR-19a on the 4, 5, 11, and 12th days post-infection (Figure 9B). As in the *in vivo* experiments, the expression patterns of *NF-*κ*B* (Figure 9C), *TNF-*α (Figure 9D) and *MyD88* (Figure 9E) in lungs were similar to those of gga-miR-19a throughout the test stages after infection. These results were consistent with the predictions obtained by bioinformatics, and indicated that MG-infection could enhance the expression of gga-miR-19a, *NF-*κ*B, TNF-*α and *MyD88*, but inhibited that of *ZMYND11*.

### gga-miR-19a regulates the NF-κB signaling pathway in DF-1 cells

**Figure 10:**
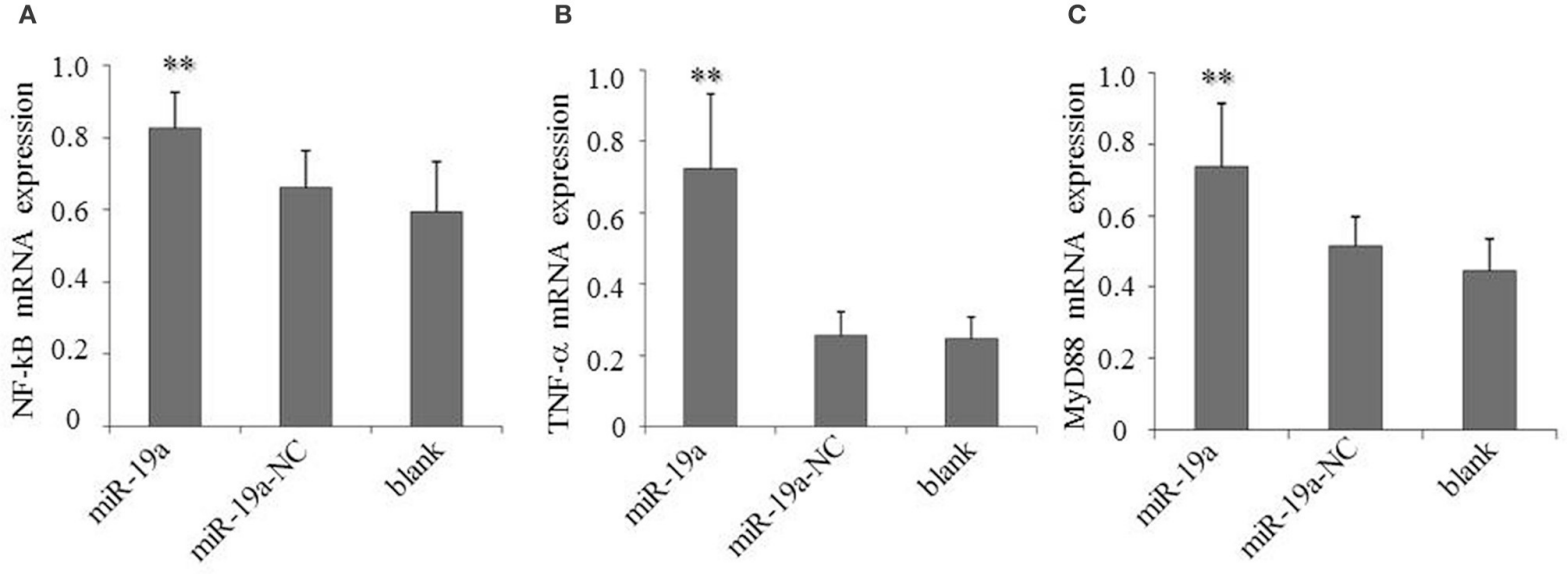
**Over-expression of gga-miR-19a activates *NF-*κ*B, TNF-*α and *MyD88* in DF-1 cells transfected with gga-miR-19a or the negative control**. A mock transfection was used as a blank. At 48 h post-transfection, the expression of *NF-*κ*B* **(A)**, *TNF-*α **(B)**, and *MyD88* **(C)** were measured by RT-qPCR. A mock transfection was used as the blank. All samples were normalized to *GAPDH*. All values are represented as the mean ± SD of three independent experiments in triplicate. Significant differences are denoted as ^**^P < 0.01.

**Figure 11:**
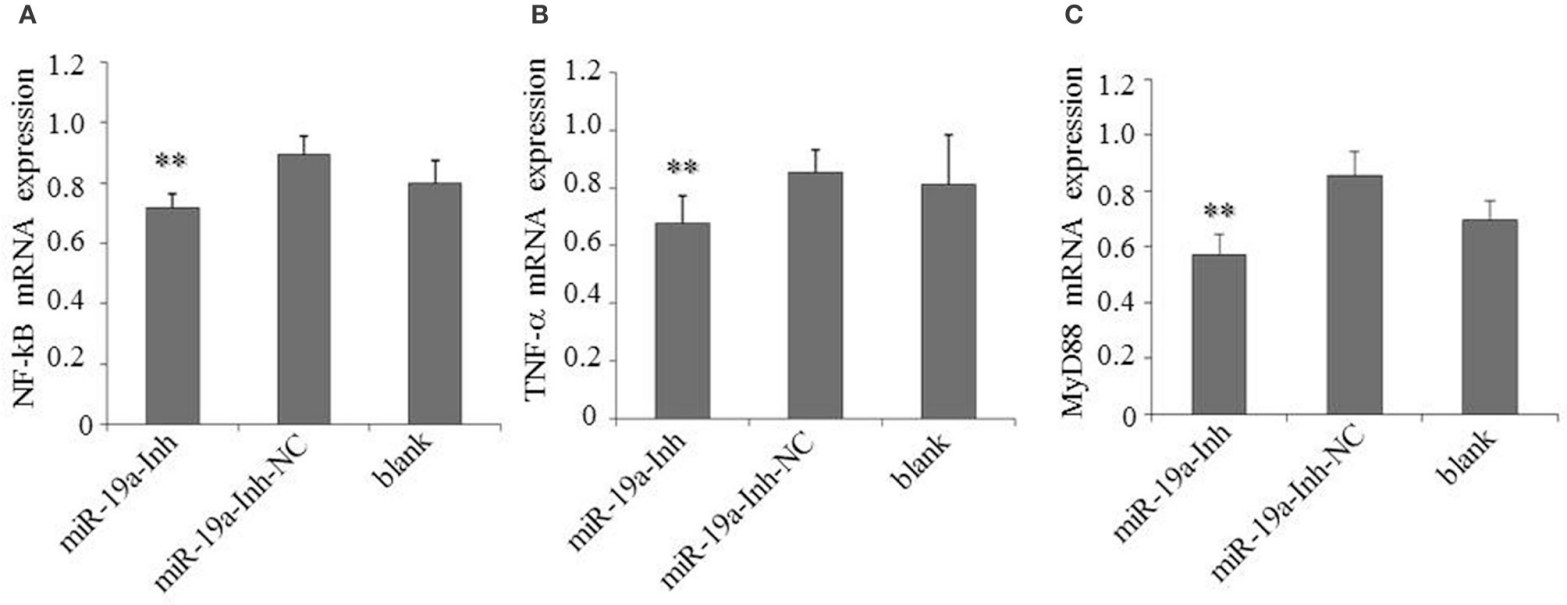
**Inhibition of gga-miR-19a reduces *NF-*κ*B, TNF-*α and *MyD88* expression**. DF-1 cells were transfected with miR-19a -Inh or the negative control. At 48 h post-transfection, the expression of *NF-*κ*B* **(A)**, *TNF-*α **(B)** and *MyD88* **(C)** were measured by RT-qPCR. A mock transfection was used as the blank, and the expression of *GAPDH* was used as a loading control. All values are represented as the mean ± SD of three independent experiments in triplicate. Significant differences are denoted as ^**^P < 0.01.

To determine the effects of gga-miR-19a on NF-κB activity, DF-1 cells were transfected with gga-miR-19a, miR-19a-NC, miR-19a-Inh or miR-19a-Inh-NC for 48 h. The over-expression of gga-miR-19a, but not miR-19a-NC, resulted in a significant increase in *NF-*κ*B, TNF-*α, and *MyD88* expression (Figures 10A–C). Conversely, *NF-*κ*B, TNF-*α, and *MyD88* expression were greatly inhibited by the over-expression of miR-19a-Inh (Figures 11A–C). These results suggest that gga-miR-19a positively regulates the activity of the NF-κB signaling pathway in inflammation by inhibiting *ZMYND11* expression.

## Discussion

It is well documented that miRNAs are critical regulators of gene silencing that exert their function through suppressing translation or/and promoting degradation of targeted mRNAs. They have important regulatory functions in multiple cellular processes, including immune responses, cellular differentiation, cellular proliferation, cell apoptosis, and inflammation (Ambros, 2004; Bartel, 2004, 2009). Accumulating evidence indicates that microbial infections can alter the expression of cellular miRNAs in chickens (Wang et al., 2009; Wang Y. S. et al., 2013; Lee et al., 2012; Lian et al., 2012; Li X. et al., 2014). Moreover, the miR-17-92 cluster (miR-17, miR-18a, miR-19a, miR-20a, miR-19b-1 and miR-92-1) has been reported to be frequently over-expressed in human cancers and has oncogenic activity (He et al., 2005; Mendell, 2008). Our previous deep sequencing results showed that some host miRNAs were aberrantly expressed, and gga-miR-19a was up-regulated in lungs of MG-infected SPF chicken embryos (unpublished lab data). Consistent with these results, this study found that gga-miR-19a expression was significantly increased in MG-infected DF-1 cells (Figure 8A) and chicken embryonic lungs on the 4, 5, 11, and 12th days post-infection (Figure 9A). Moreover, we identified *ZMYND11* as the target gene of gga-miR-19a (Figure 2). The *ZMYND11* gene showed opposite expression patterns in DF-1 cells and the lung tissues (Figures 8B, 9B.) These results also confirm that gga-miR-19a was up-regulated in cells and tissues infected by MG and could directly and negatively regulate *ZMYND11* expression by binding to the 3′UTR of *ZMYND11* mRNA.

*Mycoplasma* causes severe inflammation in humans and animals and induces a series of pro-inflammatory cytokines in a variety of cell types (Muzio et al., 2000; Barton and Medzhitov, 2003; Beutler, 2004). *Mycoplasma fermentans* lipoproteins trigger inflammatory responses via activation of TLR2 and TLR6, followed by activation of NF-κB (Takeuchi et al., 2000; Nishiguchi et al., 2001). In our previous study, TLR2 and TLR6 were up-regulated upon MG infection. This event was followed by up-regulation of the downstream NF-κB-mediated inflammatory responses (Tian et al., 2016).

The NF-κB signaling pathway is extensively involved in inflammatory responses to microbial infections (Hayden and Ghosh, 2011; Newton and Dixit, 2012). It promotes pro-inflammatory cytokines expression (e.g., TNF-α, IL-6 and IL-8) and plays an important role in the regulation of innate and adaptive immunity (Gao et al., 2014; Giles et al., 2016; Yang and Wang, 2016). These host defense mechanisms are highly conserved, including induction of cell proliferation to protect the host from infection (Lemaitre and Hoffmann, 2007; Panayidou et al., 2014). A number of miRNAs are known to participate in the regulation of the NF-κB signaling pathway at multiple steps, thus affecting the outcome of microbial infection (Lecellier et al., 2005; Ma et al., 2011b). miR-146 is an immune system regulator and plays a key role in regulating different types of diseases (Jopling et al., 2005). For example, miR-146a down-regulates the expression of TRAF6 and IRAK1 to suppress the activity of the NF-κB signaling pathway and has a key role in suppressing tumorigenesis and tumor progression by inhibiting tumor cell migration and invasion (Taganov et al., 2006). In HCV- or HIV-infected cells, the up-regulation of miR-155 and miR-21 represses the NF-κB signaling pathway (Houzet et al., 2008; Marquez et al., 2010; Zhang Y. et al., 2012). During HIV infection, the down-regulation of miR-16 results in the activation of the NF-κB signaling pathway, thus enhancing immune responses (Li et al., 2010). miR-301a promotes the activation of the NF-κB signaling pathway by down-regulating NF-κB inhibiting factors (Lu et al., 2011). miR-9 inhibits ovarian and gastric cancer cell growth through modulation of the NF-κB signaling pathway (Guo et al., 2009; Wan et al., 2010; Wang et al., 2010). miR-19 regulates the activity of the NF-κB signaling pathway to produce pro-inflammatory cytokines in inflammation (Gantier et al., 2012). Earlier studies have shown that ZMYND11 negatively regulated Epstein-Barr virus latent membrane protein 1-mediated NF-κB activation and then enhanced IL-6 expression (Ikeda et al., 2009, 2010). In this study, analysis using DAVID bioinformatics resources 6.7 indicated that *ZMYND11* could negatively regulate the NF-κB signaling pathway in chickens. In the *in vivo* experiments, MG infection resulted in a significant increase of gga-miR-19a, *NF-*κ*B, TNF-*α *and MyD88* expression but a dramatic decrease in *ZMYND11* expression (Figures 9A–E). In the *in vitro* experiments, the over-expression of gga-miR-19a resulted in a significant increase in *NF-*κ*B, TNF-*α and *MyD88* expression at mRNA levels in DF-1 cells (Figures 10A–C), whereas gga-miR-19a inhibitor drastically inhibited *NF-*κ*B, TNF-*α and *MyD88* expression at the mRNA level (Figures 11A–C). However, ZMYND11 showed the opposite pattern of expression in DF-1 cells (Figures 3B, 4B). Our results suggest that gga-miR-19a might play a critical regulatory role in activating the NF-κB signaling pathway to produce pro-inflammatory cytokines by suppressing ZMYND11 expression. gga-miR-19a might play an important role in the pathogenesis of MG infection by regulating the MyD88/NF-κB signaling pathway.

Activation of NF-κB results in the production of pro-inflammatory cytokines and affects cell proliferation (Gao et al., 2014; Yang and Wang, 2016). NF-κB promotes melanoma cell proliferation via miR-7-5p (Giles et al., 2016). The up-regulation of miR-181a activates the NF-κB signaling pathway to promote colorectal cancer cell proliferation and increase host anti-infection activity (Hai et al., 2016). The significant up-regulation of miR-301a enhances NF-κB expression to promote cell proliferation, migration and invasion in human hepatocellular carcinoma (Xie et al., 2015). gga-miR-221/222 suppresses the cell cycle of T-cell lymphoma in Marek's disease (MD) (Lambeth et al., 2009), and gga-miR-181a and gga-miR-26a inhibit proliferation of Marek's disease lymphoma cells (Li X. et al., 2014; Lian et al., 2015). The miR-17-92 cluster that is over-expressed in ESCC improves esophageal cellular proliferation both *in vitro* and *in vivo* and enhances the cell cycle progression in tumor cells (Hayashita et al., 2005; Liu et al., 2011). The up-regulation of miR-19a and miR-19b promotes cervical carcinoma cell proliferation and invasion by targeting CUL5 (Xu et al., 2012). We found that MG infection inhibits mitosis by blocking the transition from the G1 phase to the S and G2 phases in DF-1 cells (Figures 6, 7). The over-expression of gga-miR-19a accelerated the cell cycle progression to promote proliferation by enhancing the percentages of DF-1cells in the S and G2 phases (Figure 6), whereas the gga-miR-19a inhibitor repressed proliferation of the DF-1 cells by inducing the G1 phase arrest (Figure 7). Together, the results suggest that the up-expression of gga-miR-19a inhibits MG infection by improving cell proliferation through inducing the transition from the G1 phase to the S and G2 phases.

In summary, our results strongly suggest that the up-regulation of gga-miR-19a represses the expression of ZMYND11 in MG-infected cells and tissues, which in turn activates the NF-κB signaling pathway and promotes cell proliferation and the expression of pro-inflammatory cytokines to defend against MG infection. Furthermore, gga-miR-19a and its target gene ZMYND11are potential diagnostic biomarkers and therapeutic targets in the prevention and treatment of mycoplasmosis.

As both gga-miR-19a and ZMYND11 are highly conserved in a wide range of species, including human, dog, cow, chimpanzee, mouse, rat, monkey and platypus (Figure 1C), the results will provide valuable insights into the relationship between miRNAs and target genes in other species.

## Author contributions

QH and YZ, collection, assembly and analysis of the data, manuscript writing, and data analysis; ZW, discussion and manuscript revision; YH, data analysis; DB, JS, and XP, design, manuscript editing and revision. All authors read and approved the final manuscript for publication.

## Funding

This study was funded by the National Natural Science Foundation of China (Grant No. 31070154 and 31270216).

### Conflict of interest statement

The authors declare that the research was conducted in the absence of any commercial or financial relationships that could be construed as a potential conflict of interest.
